# Ruthenium-Catalyzed Selective Hydrogenation of bis-Arylidene Tetramic Acids. Application to the Synthesis of Novel Structurally Diverse Pyrrolidine-2,4-diones

**DOI:** 10.3390/molecules16076116

**Published:** 2011-07-20

**Authors:** Christos S. Karaiskos, Dimitris Matiadis, John Markopoulos, Olga Igglessi-Markopoulou

**Affiliations:** 1 Laboratory of Organic Chemistry, Department of Chemical Engineering, National Technical University of Athens, Zografos Campus, Athens 15773, Greece; Email: chriskarsp@gmail.com (C.S.K.); matiadis@mail.ntua.gr (D.M.); 2 Laboratory of Inorganic Chemistry, Department of Chemistry, University of Athens, Panepistimio-polis, Athens 15771, Greece; Email: jmmarko@chem.uoa.gr

**Keywords:** hydrogenation, homogeneous catalysis, ruthenium catalysts, BINAP, arylidenetetramic acid

## Abstract

Catalytic hydrogenation of 3,5-bis-arylidenetetramic acids, known for their biological activity, has been developed. The chemoselective ruthenium-catalyzed reduction of the exocyclic carbon-carbon double bonds on pyrrolidine-2,4-dione ring system, containing other reducible functions, has been investigated. Depending on the substrate the yield of the hydrogenation process can reach up to 95%. The structural elucidation has been established using NMR and HRMS spectral data.

## 1. Introduction

**Figure 1:**
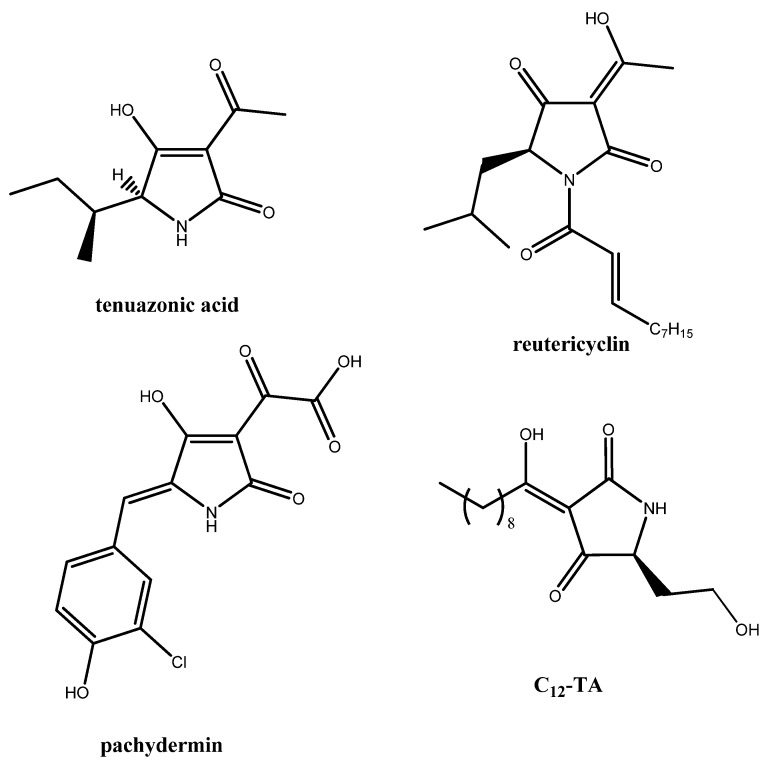
Naturally occurring tetramic acids.

Tetramic acids (TAs) containing the pyrrolidine-2,4-dione ring system are a group of naturally occurring products which possess a broad spectrum of biological and pharmaceutical activities, including antibiotic, antiviral, cytotoxic and anti-HIV agents [1]. Representative examples of tetramic acids and derivatives are tenuazonic acid [2], melophlins [3], reutericyclin [4], pachydermin [5] and dihydromaltophilin (HSAF) [6] (Figure 1). Recently, it was discovered that the Gram-negative bacterium *Pseudomonas aeruginosa* produces the antibiotic C12-TA from one of its “quorum sensing” molecules, which may also serve as a lead compound for the development of antimicrobial therapeutics [7]. Additionally, complete activities of the enzymes polyketide synthases (PKSs) and non ribosomal peptide synthases (NRPSs) have been reconstituted through the biosynthesis of the functionalized 3-acyl tetramic acid named praespyridone [8]. Moreover, tetramic acids bearing an arylidene or alkylidene group at C-5 of the heterocyclic ring have been reported as antagonists at the glycine site on *N*-methyl-D-aspartate (NMDA) receptor [9]. Consequently, multifunctionalized pyrrolidine-2,4-diones are considered as “privileged scaffolds” for the construction of a variety of biologically active nitrogen heterocycles. 

Our research group has been involved to the synthesis and structure elucidation of substituted tetramic acids with appropriate functionalities at C-3 and C-5 positions [10,11]. In this work, we are interested in the chemoselective homogeneous catalytic hydrogenation of the olefinic bond of 3-arylidene group on the pyrrolidine-2, 4-dione nucleus, over other present reducible functions, using a ruthenium-catalyst.

Homogeneous hydrogenation constitutes an important synthetic procedure and is one of the most extensively studied reactions in homogeneous catalysis. The impressive development of coordination and organometallic chemistry has allowed the preparation of a wide variety of soluble metal complexes active as homogeneous hydrogenation catalysts under mild conditions [12,13].

The selective reduction of carbon-carbon double bonds is a very important transformation in synthetic organic chemistry. Additionally, the enantioselective hydrogenation of prochiral substances bearing an activated group, such as an ester, an acid or an amide has been developed for a wide range of transformations from the laboratory to the preparation of fine chemicals and pharmaceuticals in the industry production [14,15,16].

Early advances in chemoselective olefin hydrogenation were dominated by the introduction of homogeneous transition metal complexes [17,18,19]. Many of them allow the preferential reduction of carbon-carbon double bonds over a coexisting C=O functionality [20,21,22,23,24,25].

Herein, we present a new protocol for the synthesis of 3, 5-arylidene-pyrrolidine-2,4-diones, and the chemoselective ruthenium-catalyzed hydrogenation of exocyclic substituted C=C bonds. The focus of this work will be on selective catalytic hydrogenation of double-double carbon bonds in substrates containing other reducible functions. 

## 2. Results and Discussion

**Scheme 1:**
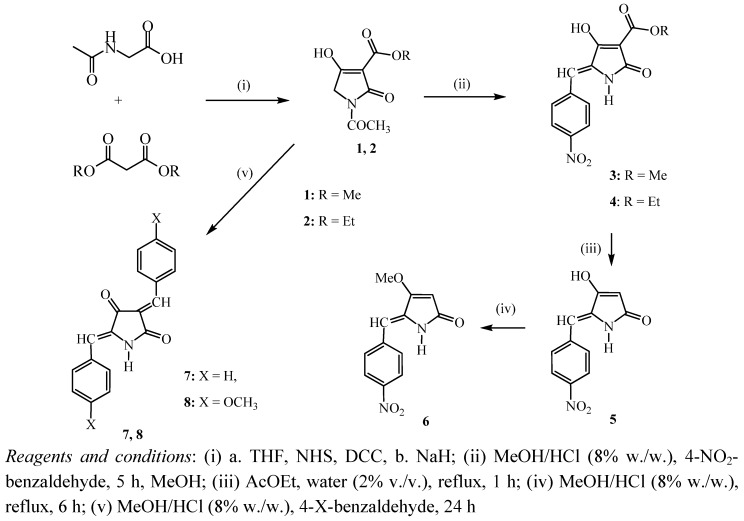
Synthesis of hydrogenation substrates.

The proposed methodology allows the construction of the 5-arylidene-pyrrolidine-2,4-dione motif in three stages (Scheme 1): (i) The synthesis of the *N*-substituted tetramic acids **1**,**2**, which can be achieved in a one-pot reaction, starting from *N*-acetylglycine [26], (ii) the production of 5-arylidene-3-alkoxycarbonyl tetramic acids **3**,**4** [27] and (iii) the elimination of the 3-alkoxycarbonyl group (Scheme 1) [28]. All these stages occur with high yield and excellent purity. Also, the 3, 5-bisarylidene tetramic acids **7**,**8** can be derived in one stage, starting from the 3-alkoxycarbonyltetramic acids by treating with a substituted benzaldehyde bearing an electron donor group.

**Figure 2:**
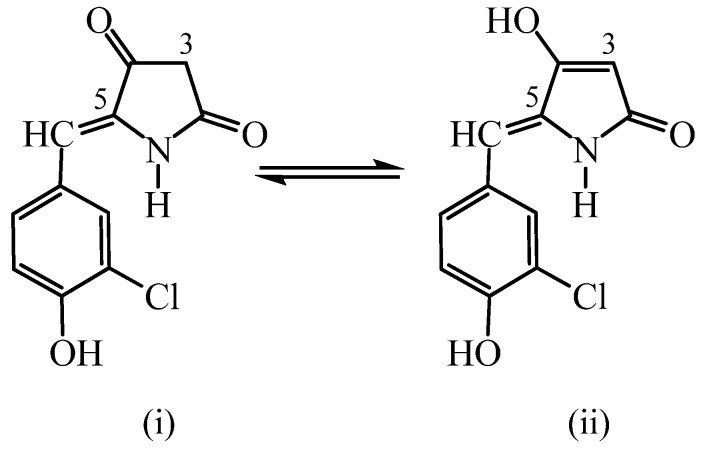
Z-configuration of the exocyclic double bond.

Comparison of the NMR spectral data of the novel 5-arylidenetetramic acids with the natural products mentioned in Figure 2 [5] verifies that they also possess the *Z*-configuration of the exocyclic double bond. 

**Figure 3:**
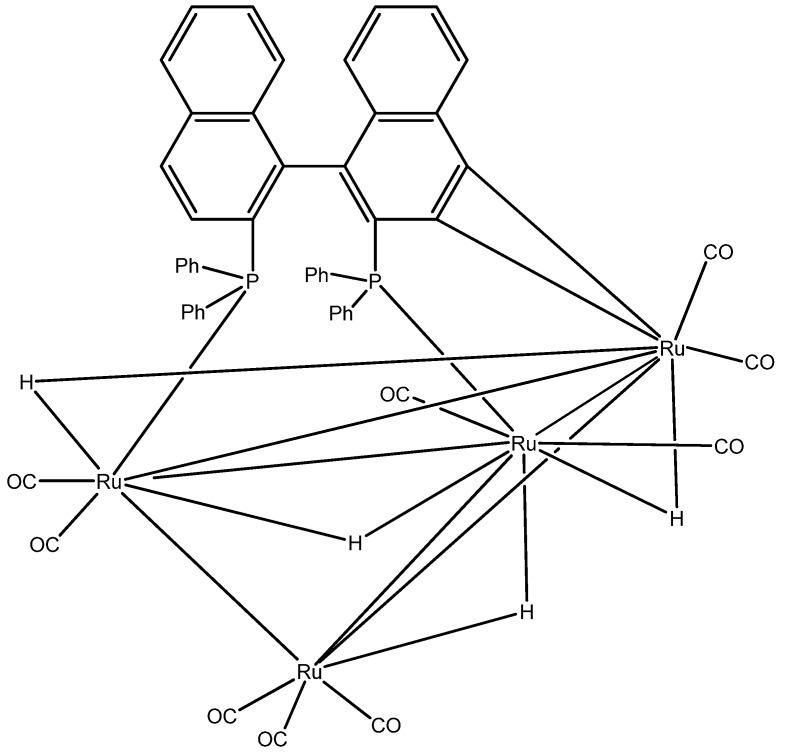
Ruthenium catalyst **14**.

Catalytic hydrogenation processes using ruthenium complexes in homogeneous phase have been widely investigated [29,30,31]. Catalysts based on Ru complexes can activate the hydrogenation of both carbonyl and olefin double bonds [14,31]. The chemoselectivity depends on the Ru complex ligands, the substrate and the reaction conditions. For the investigation of these parameters we employed: (i) different structure substrates, (ii) wide range of reaction conditions, 40–100 °C, 20–60 bar, with a ratio of substrate / catalyst of 100:1, and (iii) a catalyst structured with four Ru cores, the Η_4_Ru_4_(CO)_9_[μ_3_-(S)-BINAP] complex **14** (Figure 3). This catalyst, prepared by Professor Sergey Tunik [32] proved to be efficient on chemoselective reduction of C=C bond over a coexisting C=O group.

The solvents employed in the proposed strategy can be divided in two categories:

(1). Polar solvents (methanol, ethanol) and
(2). Non-polar solvents (tetrahydrofuran, dichloromethane)

These experimental results are summarized in Table 1. It is known that the pyrrolidine-2,4-dione nucleus ring is stable, and further stabilization occurs by the introduction of an extended conjugated double bond system (e.g. benzaldehyde, Scheme 1), It is, therefore, expected that the hydrogenation reaction conditions should be more intense than those of non conjugated systems reported in the literature [33].

**Table 1:** Hydrogenation results for compound **7 **to give **9 **and **12**.

Entry	S/C	Solvent/DCM	Temp	Press	Time	Conversion% ^a^	
			(°C)	(bar)	(h)	9	12
1	250	MeOH (24:1)	100	60	20	4.7	94.2
2	500	EtOH (24:1)	100	60	19	94.0	5.9
3	250	MeOH (24:1)	60	60	20	78.8	21.1
4	493	THF (20:1)	100	60	20	94.4	5.5

**Figure 4:**
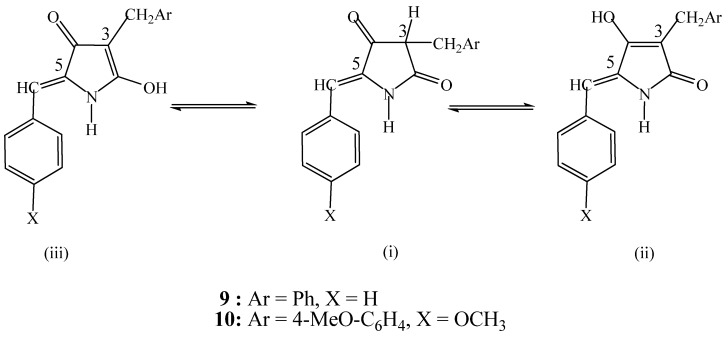
Keto-enol tautomerism of compounds **9** and **10**.

**Scheme 2:**
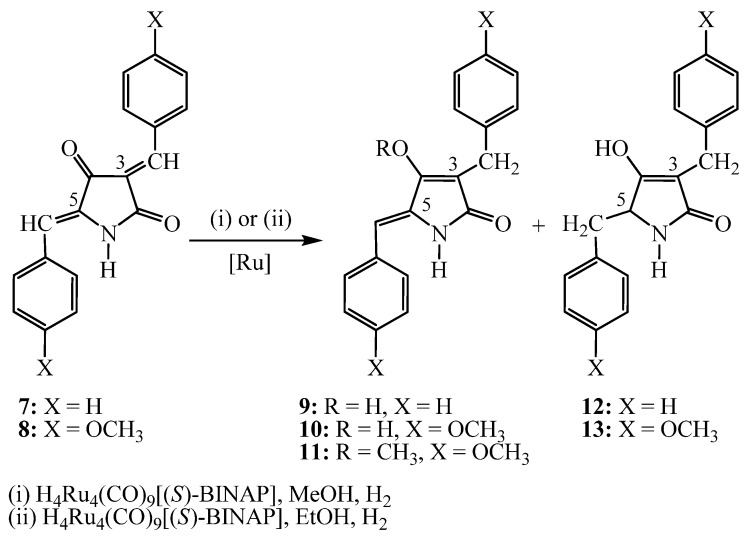
Hydrogenation of compounds **7 **and **8**.

The hydrogenation of 3,5-bisarylidene tetramic acids **7** and **8** occurs in two stages in high yields (Scheme 2, Table 1 and Table 2, entries 1–14). It is noteworthy that the hydrogenated products at the 3-position, **9** and **10**, do not exhibit a chiral center at C-3. The best outcome may be attributed to the racemization of the chiral carbon center C-3 through enolization [34] (Figure 4), and the prevalence of the enol tautomers. This is a well established phenomenon on the structure elucidation of functionalized pyrrolidine-2, 4-diones, on the basis of experimental and theoretical research.

**Table 2:** Hydrogenation results for compound **8** to give **10** and **13**, based on the reaction scheme 2.

Entry	S/C	Solvent/DCM	Temp	Press	Time	Conversion% ^a^	
			(°C)	(bar)	(h)	10	13
5	120	MeOH (24:1)	100	60	20	24.8	73.9
6	405	MeOH (24:1)	100	60	20	44.0	51.6
7 ^b^	405	MeOH (24:1)	100	60	41	28.8	47.5
8	429	MeOH (24:1)	80	60	20	13.8	84.4
9	429	MeOH (24:1)	60	60	20	93.3	2.9
10	227	MeOH (24:1)	40	60	20	86.2	3.7
11	405	MeOH (24:1)	80	40	20	34.0	64.7
12	405	MeOH (24:1)	80	20	20	53.2	45.7
13	405	EtOH (24:1)	80	60	20	95.0	5.0
14	700	THF (20:1)	100	60	20	25.4	8.9

^a^ Calculated by HPLC, using a Chiralpak AS column (4.6 × 250 mm); ^b^ Compound **11** was isolated in 23.7% conversion (see Experimental).

This statement has been verified by comparison of the ^1^H NMR and MS spectral data for these products. Only the “enolic tautomers” of the products **9**, **10**, **12** and **13** were isolated. In the same solvent the optimal yields of compounds **10** (entry 9) and **13** (entry 8) were achieved at 60 °C and 80 °C, respectively.

The variation of the applied hydrogen pressure is less effective compared to the temperature, with respect to the yield of **10** and **13**, when the temperature is constant, for the same solvent (entries 11, 12). However, higher pressure favors the formation of **13**.

The purification of the solvents is important in order to maintain neutral conditions during the hydrogenation reactions. The Ru cluster is very sensitive to water and oxygen. For this reason the solvents are dried and sonicated prior to their use. The solvent has the greatest impact, among all the reaction parameters, on the hydrogenation result. Further examination of the solvent effect revealed that methanol favors the formation of compounds **12** and **13** and ethanol predominantly leads to the formation of compounds **9** and **10** (entries 2, 13, Table). Tetrahydrofuran gives good results only in the case of substrates **7** and **8** (entries 4 and 14, respectively). Dichloromethane is employed for the introduction of the Ru complex in the reaction mixture, and used in small amounts, in every case. Using only dichloromethane as the reaction solvent for the hydrogenation of compound **10**, we cannot obtain product **13**. The novel substrates and hydrogenation products were characterized by micro analysis, HRMS, ^1^H- and ^13^C-NMR spectroscopy [35,36,37,38]. 

## 3. Experimental

### 3.1. Preparation of solvents and substrates

Purification of methanol, ethanol and dichloromethane solvents included distillation from CaH_2_ under a nitrogen atmosphere. Tetrahydrofuran was distilled from Na/Ph_2_CO, in an argon atmoshpere. All manipulations were performed using standard Schlenk techniques. Prior to the reaction all solutions were sonicated for 20 min to remove oxygen and other gases, at room temperature and under an argon atmosphere. Flash column chromatography was carried out on Macherey–Nagel 0.063–0.2 mm/70–230 mesh silica gel.

The hydrogenation reaction took place in an Autoclave Engineers’ 300 mL batch reactor. The reactor was equipped with a glass liner, two thermocouples (for the measurement of the process and the heater temperature), a pressure gauge, and a PID control tower (CT-1000). The stainless steel mechanical stirrer, that can go up to 2,500 rpm, was insulated with Teflon. The autoclave has a wide operational range, from −50 °C to 250 °C, and from 1 bar to 200 bar. 

The NMR spectra were recorded on a Varian Gemini 2000 300 MHz spectrometer. MS spectra were recorded on a Micromass LCT Mass Spectrometer (Chemistry Department, University of Liverpool, Liverpool, UK). Melting points were determined on a Gallenkamp MFB-595 melting point apparatus and are uncorrected.

### 3.2. Synthesis of 5-arylidenetetramic acids

*5-Arylidene(4'-nitro)tetramic acid *(**5**) [39]: A mixture of 3-ethoxycarbonyl-5-arylidene(4'-nitro) tetramic acid (2.0 mmol) and ethyl acetate (60 mL) in water (1.2 mL), was heated under reflux for 1 h. After cooling to room temperature the solution was dried over anhydrous Na_2_SO_4_, and filtered off. The filtrate was evaporated under reduced pressure and dried *in vacuo* to afford the compound **5**. The solid was washed several times with dry diethyl ether. Yellow solid (from ethyl acetate), yield 74%; mp 238–239 °C (decomposed); ^1^H-NMR (DMSO-*d*_6_): δ 3.24/5.06 (2s, 2H/1H, 3-H), 6.26/6.32 (2s, 1H/1H, 6-H), 7.78/7.83 (2d, 2H/2H, b- and c-H, *J* = 8.7 Hz), 8.16/8.18 (2d, 2H/2H, d- and e-H, *J* = 8.7 Hz), 9.73/11.28 (2s, 1H/1H, NH), 12.03 ppm (s, 1H, OH); ^13^C-NMR (DMSO-*d*_6_): δ 93.0 (C-3), 101.7/102.4 (C-6), 123.7 (C-d, C-e), 129.8/130.1 (C-b, C-c), 136.6/136.9 (C-5), 140.4/141.3 (C-a), 145.6/146.0 (C-f), 165.7/172.1 (C-2), 173.0/195.5 ppm (C-4); HRMS calcd. for [C_11_H_8_N_2_O_4_+23Na]^+^: 255.0382, found: 255.0397.

*5-Arylidene(4'-nitro)-4-methoxytetramic acid* (**6**): 5-Arylidene(4'-nitro)tetramic acid (1.5 mmol) was refluxed for 6 h in a solution of 8% hydrochloric acid in methanol (prepared by addition of acetyl chloride (2 mL) in anhydrous methanol (15 mL) until it was dissolved), and then the reaction mixture was stirred overnight at room temperature. The resulting solid was filtered off and washed several times with diethyl ether. Dark yellow solid (from methanol), yield 82%; mp 275–276 °C; ^1^H-NMR (DMSO-*d*_6_): δ 3.91 (s, 3H, 7-H), 5.45 (s, 1H, 3-H), 6.29 (s, 1H, 6-H), 7.80 (d, 2H, b- and c-H, *J* = 8.7 Hz), 8.17 (d, 2H, d- and e-H, *J* = 8.4 Hz), 10.02 ppm (s, 1H, NH); ^13^C-NMR (DMSO-*d*_6_): δ 58.8 (C-7), 93.2 (C-3), 102.9 (C-6), 123.7 (C-d, C-e), 130.0 (C-b, C-c), 135.1 (C-5), 140.9 (C-a), 145.8 (C-f), 167.2 (C-2), 172.0 ppm (C-4); HRMS calcd. for [C_12_H_10_N_2_O_4_+23Na]^+^: 269.0538, found 269.0548.

*3, 5-Bis(benzylidene)tetramic acid *(**7**) [40]: 3-Ethoxycarbonyltetramic acid (1.0 mmol) was stirred in a solution of 8% hydrochloric acid in methanol, prepared from the addition of acetyl chloride (1 mL) in anhydrous methanol (7 mL) until it was dissolved. Benzaldehyde (3.0 mmol) was added in the solution and the reaction mixture was refluxed for 24 h, and then left under stirring overnight. The precipitated was filtered off, washed several times with diethyl ether and dried *in vacuo* for 5 h, to afford the corresponding product, as a purple solid, yield 63%; mp 213–214 °C; ^1^H-NMR (DMSO-*d*_6_): δ 6.47 (s, 1H, 6-H), 7.30–7.44 (m, 3H, j-, k- and l-H), 7.56–7.68 (m, 5H, h-, i-, d-, e- and f-H), 7.88/7.91 (2s, both, 1H, 7-H), 8.52–8.59 (m, 2H, b- and c-H), 11.17 ppm (s, 1H, NH); ^13^C-NMR (DMSO-*d*_6_): δ 106.6 (C-6), 122.4 (C-3), 128.2 (C-f, C-l), 128.7 (C-b, C-c), 128.8 (C-h, C-i), 129.5 (C-j, C-k), 129.6 (C-d, C-e), 133.2 (C-5), 133.5 (C-a), 133.7 (C-g), 148.1 (C-7), 166.9 (C-2), 184.1 ppm (C-4); HRMS calcd. for [C_18_H_13_NO_2_+23Na]^+^: 298.0844, found: 298.0846 and calcd. for [C_18_H_13_NO_2_+23Na+MeOH]^+^: 330.1106, found: 330.1102.

*3, 5-Bis[arylidene(4'-methoxy)]tetramic acid* (**8**) [39]: 3-Ethoxycarbonyltetramic acid (1.0 mmol) was stirred in a solution of 8% hydrochloric acid in methanol, prepared from the addition of acetyl chloride (2 mL) in anhydrous methanol (15 mL) until it dissolved. *p*-Methoxybenzaldehyde (3.0 mmol) was then added in the solution, the reaction mixture was refluxed for 24 h and then left under stirring overnight. The precipitated was filtered off, washed several times with diethyl ether and then dried *in vacuo* for 5 h, to afford the corresponding product as a purple solid, yield 66%; mp 239–240 °C; ^1^H- NMR (DMSO-*d*_6_): δ 3.80 (s, 3H, OCH_3_ (C-f)), 3.89/3.90 (2s, both, 3H, OCH_3_ (C-l)), 6.44 (s, 1H, 6-H), 6.97 (d, 2H, d- and e-H, J = 8.4 Hz), 7.14 (d, 2H, j- and k-H, *J* = 9.0 Hz), 7.63 (d, 2H, b- and c-H, *J* = 8.7 Hz), 7.80 (s, 1H, 7-H), 8.64 (d, 2H, h- and i-H, *J* = 9.0 Hz), 10.90 ppm (s, 1H, NH); ^13^C-NMR (DMSO-*d*_6_): δ 55.2 (OCH_3_ [C-f]), 55.8 (OCH_3_ [C-l]), 106.7 (C-6), 114.3 (C-d, C-e), 114.5 (C-j, C-k), 125.8 (C-3), 126.2 (C-5), 130.9 (C-b, C-c), 131.2 (C-h, C-i), 136.7 (C-g), 137.7 (C-a), 147.9 (C-7), 159.3 (C-f), 164.1 (C-l), 167.5 (C-2), 184.0 ppm (C-4); HRMS calcd. for [C_20_H_17_NO_4_+23Na]^+^: 358.1055, found: 358.1065.

### 3.3. Procedure for the hydrogenation reaction

The autoclave was sealed and was flushed three times with nitrogen. The substrate was dissolved in the proper solvent (methanol, ethanol or THF), and the catalyst was dissolved in DCM, both under argon atmosphere. The solutions were mixed under argon atmosphere and were sonicated for 20 min. Then the autoclave was loaded with the reaction mixture from a designated reactant inlet, under nitrogen current. The inlet was then sealed, the autoclave was flushed several times with hydrogen (pressurized with 10 bar H_2_ each time), and then pressurized to the desired pressure with hydrogen. Finally, the stirrer speed was adjusted and the temperature was set to the desired value. After the completion of the hydrogenation process the reaction vessel was cooled to room temperature and the pressure was relieved. Next the solvent was evaporated under reduced pressure to give a solid product that was washed several times with diethyl ether and was purified by silica gel chromatography (CH_2_Cl_2_/MeOH 95:5–92:8).

### 3.4. Synthesis of hydrogenation products

*3-Benzyl-5-benzylidene-4-hydroxy-3-pyrroline-2-one* (**9**) [28,40]: It was derived as a pale yellow solid (from methanol), mp 188–189 °C; ^1^H-NMR (DMSO-*d*_6_): 3.58 (s, 2H, 7-H), 6.25 (s, 1H, 6-H), 7.22 (m, 6H), 7.35 (t, 2H), 7.55 (d, 2H, *J* = 7.2 Hz), 9.48 (s, 1H, NH), 11.04 ppm (s, 1H, (4)-OH). ^13^C-NMR (DMSO-*d*_6_): 26.2 (C-7), 104.1 (C-3), 104.9 (C-6), 125.5 (C-l), 127.1 (C-f), 127.6/128.0/128.5/128.8 (C-b, C-c, C-d, C-e, C-h, C-I, C-j, C-k), 132.5 (C-5), 134.2 (C-a), 140.0 (C-g), 159.4 (C-4), 172.7 ppm (C-2). HRMS calcd. for [C_18_H_15_NO_2_+23Na]^+^: 300.1000, found: 300.0999.

*3-p-Methoxybenzyl-5-p-methoxybenzylidene-4-hydroxy-3-pyrroline-2-one *(**10**): It was derived as a pale yellow solid (from methanol), mp 189–190 °C; ^1^H-NMR (DMSO-*d*_6_): 3.49 (s, 2H, 7-H), 3.70 (s, 3H, OCH_3_ [C-l]), 3.77 (s, 3H, OCH_3_ [C-f]), 6.19 (s, 1H, 6-H), 6.82 (d, 2H, j- and k-H, *J* = 7.5 Hz), 6.92 (d, 2H, d- and e-H, *J* = 8.1 Hz), 7.14 (d, 2H, h- and i-H, *J* = 8.1 Hz), 7.49 (d, 2H, b- and c-H, *J *= 8.7 Hz), 9.37 (s, 1H, NH), 10.85 ppm (s, 1H, (4)-OH); ^13^C-NMR (DMSO-*d*_6_): 25.5 (C-7), 55.0 (OCH_3_ [C-l]), 55.2 (OCH_3_ [C-f]), 104.4 (C-3), 105.1 (C-6), 113.6 (C-j, C-k), 114.2 (C-d, C-e), 127.0 (C-a), 129.1 (C-b, C-c), 130.5 (C-h, C-i), 130.9 (C-g), 132.3 (C-5), 157.4 (C-4), 158.6 (C-l), 159.2 (C-f), 172.9 ppm (C-2). HRMS calcd. for [C_20_H_19_NO_4_+23Na]^+^: 360.1212, found: 360.1221.

*3-p-Methoxybenzyl-5-p-methoxybenzylidene-4-methoxy-3-pyrroline-2-one* (**11**): It was derived as a pale yellow solid (from methanol), mp 184–185 °C; ^1^H-NMR (DMSO-*d*_6_): 3.71 (s, 3H, OCH_3_ [C-l]), 3.73 (s, 2H, 7-H), 3.78 (s, 3H, OCH_3_ [C-f]), 3.98 (s, 3H, OCH_3 _ [C-4]), 6.13 (s, 1H, 6-H), 6.86 (d, 2H, j- and k-H, *J* = 7.5 Hz), 6.92 (d, 2H, d- and e-H, *J* = 8.1 Hz), 7.11 (d, 2H, h- and i-H, *J* = 8.1 Hz), 7.51 (d, 2H, b- and c-H, *J* = 8.7 Hz), 9.78 ppm (s, 1H, NH); ^13^C-NMR (DMSO-*d*_6_): 26.4 (C-7), 55.0 (OCH_3_ [C-l]), 55.2 (OCH_3_ [C-f]), 59.0 (OCH_3_ [C-4]), 105.7 (C-3), 105.9 (C-6), 113.9 (C-j, C-k), 114.2 (C-d, C-e), 126.7 (C-a), 128.7 (C-b, C-c), 130.1 (C-g), 130.7 (C-h, C-i), 132.6 (C-5), 157.5 (C-4), 158.7 (C-l), 159.7 (C-f), 172.6 ppm (C-2). HRMS calcd. for [C_21_H_21_NO_4_+23Na]^+^: 374.1368, found: 374.1367.

*3, 5-Dibenzyl-4-hydroxy-3-pyrroline-2-one* (**12**): It was derived as a pale yellow solid (from methanol), mp 72–73 °C; ^1^H-NMR (DMSO-*d*_6_): 2.87–3.05 (dd, 2H, 6-H), 3.16/3.22 (2s, 2H, 7-H), 4.17 (br, 1H, 5-H), 6.75 (d, 2H, *J* = 6.9 Hz), 7.08 (d, 3H, *J* = 6.6 Hz), 7.21 (m, 5H), 7.34 (s, 1H, NH), 10.81 ppm (s, 1H, (4)-OH). ^13^C-NMR (DMSO-*d*_6_): 26.2 (C-7), 36.5 (C-6), 56.0 (C-5), 104.6 (C-3), 125.1 (C-l), 126.2 (C-f), 127.7 (C-d, C-e, C-j, C-k), 128.1 (C-4), 128.6 (C-i), 129.1 (C-h), 129.7 (C-b, C-c), 136.2 (C-a), 140.1 (C-g), 168.6 ppm (C-2). HRMS calcd. for [C_18_H_17_NO_2_+23Na]^+^: 302.1157, found: 302.1199.

*3, 5-di-p-Methoxybenzyl-4-hydroxy-3-pyrroline-2-one* (**13**): It was derived as a pale yellow solid (from methanol), mp 81–82 °C; ^1^H-NMR (DMSO-*d*_6_): 2.82–3.96 (dd, 2H, 6-H, *J* = 9.9 Hz), 3.07/3.12/3.17/ 3.23 (4s, 2H, 7-H), 3.67 (s, 3H, OCH_3_ [C-l]), 3.73 (s, 3H, OCH_3_ [C-f]), 4.11 (br, 1H, 5-H), 6.61 (s, 4H), 6.77 (d, 2H, *J *= 8.4 Hz), 7.09 (d, 2H, *J* = 8.4 Hz), 7.30 (s, 1H, NH), 10.68 ppm (s, 1H, (4)-ΟH).^ 13^C-NMR (DMSO-*d*_6_): 25.2 (C-7), 35.4 (C-6), 54.8 (OCH_3_ [C-l]), 55.0/55.1 (OCH_3_ [C-f]), 56.0 (C-5), 105.1 (C-3), 113.0/113.1 (C-d, C-e, C-j, C-k), 127.8 (C-a), 128.6 (C-b, C-c), 130.7 (C-h, C-i), 132.0 (C-g), 156.9 (C-l), 157.7 (C-f), 168.3 (C-4), 173.8 ppm (C-2). HRMS calcd. for [C_20_H_21_NO_4_+23Na]^+^: 362.1368, found: 362.1364.

## 4. Conclusions

A high efficient ruthenium-catalyzed reduction of the carbon-carbon double bonds of biologically active arylidene-pyrrolidine-2, 4-diones has been developed. The availability of the substrates and their remarkable biological evaluation should make this method attractive to synthetic chemists. The proposed methodology, therefore, represents an efficient and novel catalytic approach to prepare novel pharmaceutically and biologically valuable specifically functionalized tetramic acids. Further investigation into related processes and the enantioselective identification are under investigation by our group.
